# Research on Water and Fertilizer Use Strategies for Silage Corn Under Different Irrigation Methods to Mitigate Abiotic Stress

**DOI:** 10.3390/plants15020228

**Published:** 2026-01-11

**Authors:** Delong Tian, Yuchao Chen, Bing Xu, Guoshuai Wang, Lingyun Xu

**Affiliations:** 1 Institute of Water Resources for Pastoral Area Ministry of Water Resources, Hohhot 010020, China; mkstdl@126.com (D.T.); nmxubing@163.com (B.X.); imau_wgs@163.com (G.W.); 2 Yinshanbeilu Grassland Eco-Hydrology National Observation and Research Station, China Institute of Water Resources and Hydropower Research, Beijing 100038, China; 3 School of Water Resources and Hydro-Electric Engineering, Xi’an University of Technology, Xi’an 710048, China; 4 College of Water Conservancy and Civil Engineering, Inner Mongolia Agricultural University, Hohhot 010018, China

**Keywords:** water–fertilizer–thermal coupling, water and fertilizer system, drip irrigation under mulch film, shallow-buried drip irrigation, AquaCrop model

## Abstract

To reconcile the intensifying trade-off between chronic water scarcity and escalating forage demand in the Yellow River Basin, this study optimized integrated irrigation and fertilization regimes for silage maize. Leveraging the AquaCrop model, validated by 2023–2024 field experiments and a 35-year (1990–2024) meteorological dataset, we systematically quantified the impacts of multi-factorial water–fertilizer–heat stress under drip irrigation with mulch (DIM) and shallow-buried drip irrigation (SBDI). Model performance was robust, yielding high simulation accuracy for soil moisture (RMSE < 3.3%), canopy cover (RMSE < 3.95%), and aboveground biomass (RMSE < 4.5 t·ha^−1^), with EF > 0.7 and R^2^ ≥ 0.85. Results revealed distinct stress dynamics across hydrological scenarios: mild temperature stress predominated in wet years, whereas severe water and fertilizer stresses emerged as the primary constraints during dry years. To mitigate these stresses, a medium fertilizer rate (555 kg·ha^−1^) was identified as the stable optimum, while dynamic irrigation requirements were determined as 90, 135, and 180 mm for wet, normal, and dry years, respectively. Comparative evaluation indicated that DIM achieved maximum productivity in wet years (aboveground biomass yield 70.4 t·ha^−1^), whereas SBDI exhibited superior “stable yield–water saving” performance in normal and dry years. The established “hydrological year–irrigation method–threshold” framework provides a robust decision-making tool for precision management, offering critical scientific support for the sustainable, high-quality development of livestock farming in arid regions.

## 1. Introduction

As the world’s second most populous country with more than 1.4 billion people, China is facing a growing contradiction between limited water resources and growing food security needs. Although China’s total freshwater resources are among the highest in the world, its per capita share is only about 2100 m^3^—only a quarter of the world average—making it one of the world’s most water-stressed countries [1,2]. Agriculture consumes more than 60% of China’s total water intake, and this pressure is particularly prominent in the Yellow River Basin (YRB). The rigid water quota in this area often has a significant conflict with the expansion of high-water-consuming crops [3,4]. At the same time, China’s animal husbandry is undergoing a rapid transformation to high-quality and large-scale production. Silage corn has become the cornerstone of this transformation because of its high biomass yield and high energy density [5].

As a key agro-pastoral ecotone, the Yellow River irrigation area in Inner Mongolia has contributed greatly to this supply chain [6,7]. However, the region generally faces a rigid gap in water resources, high bulk density in the topsoil of some plots, and large differences in water and fertilizer conservation capacity, which seriously restricts the release of agricultural production potential. The problem of excessive irrigation and unbalanced fertilization caused by traditional water and fertilizer management depends on experience, which further causes low water use efficiency and serious fertilizer loss. It also easily causes ecological problems such as soil nutrient imbalance and threatens the sustainable development of agriculture. More importantly, there is a significant multiple stress dilemma in the region—water restriction causes water stress, resulting in insufficient water supply for maize growth; the temperature of well water is low, and direct irrigation causes the soil temperature to drop sharply to form temperature stress, which inhibits the absorption capacity of roots to water and fertilizer; the imbalance of fertilization brings about fertilizer stress, resulting in an imbalance of the soil nutrient ratio and a decrease in fertilizer utilization rate. At present, the effect of water and fertilizer under the combined action of these three stresses is not clear, and the optimization strategy of water and fertilizer for different hydrological years requires in-depth study, which makes it difficult for farmers to implement scientific water and fertilizer management, and they cannot fully tap the production potential of silage corn [8,9]. Therefore, under the goal of water-saving agriculture and ‘double carbon’, it is urgent to optimize the water and fertilizer system of silage maize by combining quantitative simulations with field experiments [10,11,12].

Crop growth models are an effective tool to quantify the interaction between water and fertilizer–crop–environment [13,14,15]. Compared with more complex models such as DSSAT [16,17] or WOFOST [18,19], AquaCrop has attracted much academic attention due to its ‘water-driven’ core engine and the balance between model ease of use and simulation accuracy [20,21]. The model is widely used in the analysis of deficit irrigation scenarios in water-shortage areas, the effect of nutrient stress on crop growth, and the optimization of farmland water and fertilizer [22,23]. However, there are still significant research gaps: the existing simulation studies rarely consider the interaction of ‘water–fertilizer–heat’ compound stress in silage maize under different drip irrigation modes in sandy soil environments. In addition, the parameter calibration and verification data in this area are insufficient, and further calibration is needed in combination with field-measured data.

In this study, a comprehensive quantitative study on the correlation of ‘water–fertilizer–heat’ triple stress was carried out in the long-term simulation framework for silage maize in the Yellow River Basin. Different from previous studies that focused on single-factor stress or general soil types [24,25], our work introduced temperature stress into the AquaCrop model to evaluate the growth dynamics of silage maize. The key is that this study constructs a decision matrix suitable for different hydrological years (wet years, normal years, and dry years) by using a 35-year meteorological dataset, which transcends the limitations of short-term field trials. This provides a mechanistic understanding of how film-mediated heat preservation and irrigation depth interact to alleviate abiotic stress.

Based on the above research gaps, the objectives of this study include the following: calibration and validation of the AquaCrop model of silage maize under different drip irrigation modes (DIM and SBDI) in the Yellow River irrigation area; quantitative analysis of the characteristics and evolution of water, fertilizer and temperature stress under different hydrological scenarios; and determination of the optimal water and fertilizer threshold that can minimize abiotic stress while maximizing yield and water use efficiency. This study can provide a scientific basis for the precise management of silage maize in arid and semi-arid areas.

## 2. Results

### 2.1. Model Calibration and Validation

The AquaCrop model was calibrated using field data from 2023. As shown in Table 1, the model demonstrated robust predictive accuracy for soil moisture, canopy cover (CC), and aboveground biomass (AGB) across both DIM and SBDI treatments. The statistical indicators revealed a high goodness-of-fit, with R^2^ ≥ 0.85 and EF ranging from 0.71 to 0.96. Specifically, the RMSE was restricted to 2.43–3.25% for soil moisture, 2.86–3.92% for CC, and 3.1–4.3% for AGB. These results verify that the calibrated model effectively captures the temporal dynamics of soil water status and crop growth, providing a reliable foundation for subsequent scenario simulations under multi-factorial stress.

The model parameters were calibrated using field test data from 2023 and validated using field test data from 2024. Some parameters of AquaCrop simulation of silage maize growth were obtained [26,27] as shown in Table 2 and Table 3.

The corrected crop parameters for silage maize were input into the corresponding modules of the model for model validation, and then validated using measured data from 2024. The validation results are shown in Table 4. The RMSE for soil moisture content evaluation of silage maize was 2.48–2.65%, EF was 0.81–0.87, and R^2^ was 0.86–0.94; the RMSE for canopy coverage evaluation was 3.14–3.75%, EF was 0.80–0.89, and R^2^ was 0.86–0.92; and the RMSE for yield evaluation was 3.2–3.9 ton·ha^−1^, EF was 0.75–0.86, and R^2^ was 0.86–0.93. The results show good simulation accuracy, indicating that the model can accurately capture the basic changes in soil moisture content, canopy coverage, and yield under shallow-buried drip irrigation and subsurface drip irrigation for silage maize, and has good predictive ability for crop production.

### 2.2. Physiological Response and Growth Dynamics Under Combined Stress

#### 2.2.1. Effects of Different Water and Fertilizer Treatments on Canopy Coverage

**Figure 1:**
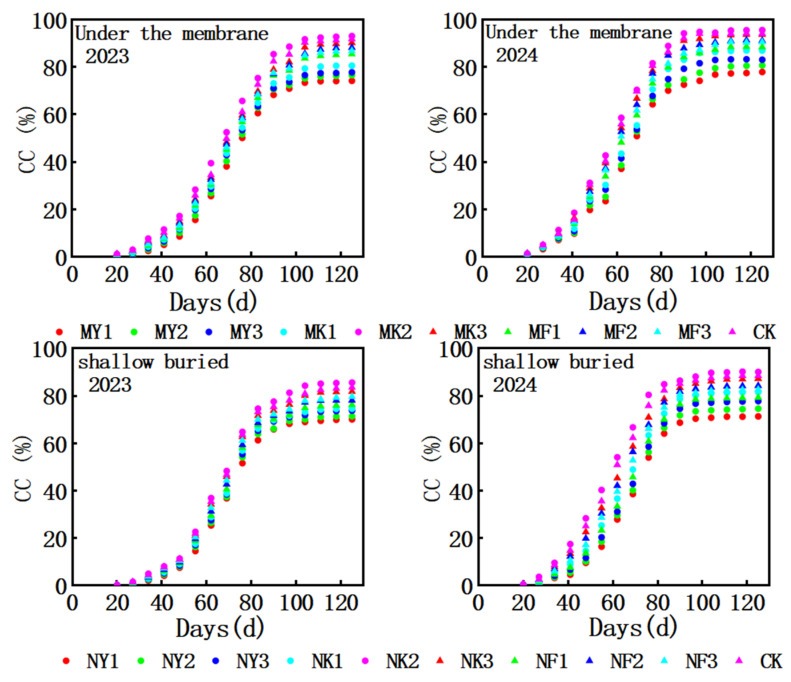
Canopy coverage maps of maize silage using different methods in 2023 and 2024. CC, canopy cover.

Figure 1 shows the changes in canopy cover of silage maize in 2023 and 2024. Crop canopy cover changed significantly with the crop’s growth and development stages, exhibiting a generally consistent growth pattern, gradually increasing in a unimodal manner. Subsurface drip irrigation significantly reduced inter-row evaporation and increased topsoil temperature, leading to rapid canopy cover growth in the early growth stages. The water–fertilizer interaction effect indicated that in 2023 and 2024, high water and high fertilizer (MY3) under mulching conditions easily induced redundant growth, resulting in excessively high CC peaks and decreased light transmittance, which was detrimental to dry matter accumulation. The MK2 treatment showed the highest canopy cover, with the difference between the highest and lowest canopy cover values over the two years ranging from 18% to 21%. In 2023 and 2024, shallow-buried drip irrigation achieved optimal results under the medium-water and -fertilizer (NK2) treatment, with the difference between the highest and lowest canopy cover values over the two years ranging from 19% to 20%.

#### 2.2.2. Effect of Accumulated Temperature on Aboveground Dry Matter Accumulation Under Different Water and Fertilizer Treatments

**Figure 2:**
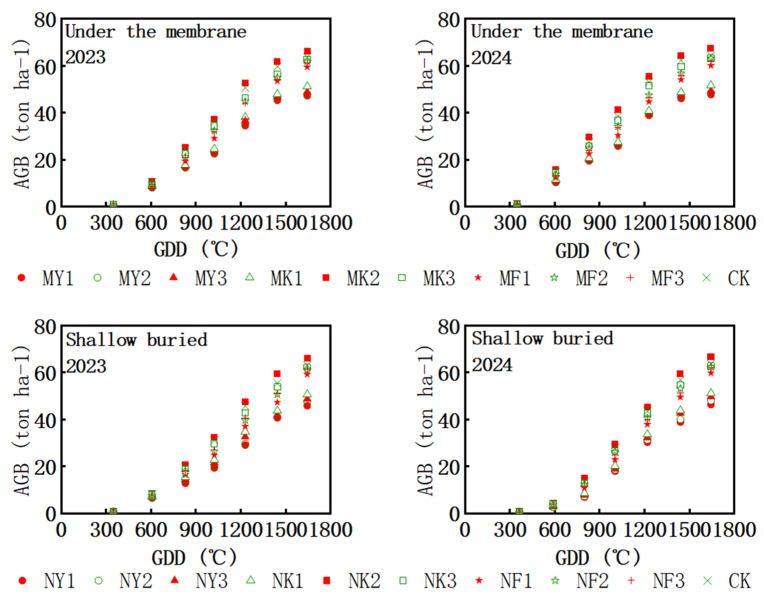
Dry matter quality map of silage corn aboveground biomass in different drip irrigation modes in 2023 and 2024. GDD, growing degree days; AGB, aboveground biomass.

The aboveground dry matter accumulation of silage maize under different water and fertilizer systems is shown in Figure 2. The aboveground dry matter accumulation of silage maize increased significantly with the increase in effective accumulated temperature. In 2023 and 2024, the accumulation of aboveground dry matter under MK2 treatment was the highest, at 66.0 ton ha^−1^ and 66.7 ton ha^−1^, respectively. The dry matter mass above the ground entered the linear growth period when the effective accumulated temperature reached 900 °C·d, which was 15–18% higher than that of shallow-buried drip irrigation under the same effective accumulated temperature. However, the growth rate of dry matter mass above ground slowed down after the effective accumulated temperature >1400 °C·d of high-water and high-fertilizer treatment (MY3), indicating that the coupling of “water–fertilizer–heat” under mulching conditions could easily trigger the physiological upper limit. The accumulation rate of aboveground dry matter in shallow drip irrigation reached its maximum between 1025~1230 °C in the MK2 treatment and 1000~1220 °C in the MK3 treatment in 2023 and 2024, respectively, which was 73.3 kg ha^−1^/°C and 73.73 kg ha^−1^/°C. Although the early accumulated temperature response was delayed, the water and fertilizer supplies were stable in the middle and late stages, and the dry matter mass on the ground continued to accumulate to the effective accumulated temperature of 1600 °C·d without attenuation.

#### 2.2.3. Analysis of Water and Fertilizer Use Efficiency Under Different Water and Fertilizer Treatments

Table 5 shows that during the two-year experiment, the MK2 and NK2 treatments exhibited significant yield advantages in both mulched and unmulched conditions. Even with irrigation amounts of 135 mm and fertilizer application rates of 555 kg·ha^−1^, these treatments showed no significant difference in yield compared to the control (CK). In 2023, the highest water use efficiency (UHE) values for both mulched and unmulched conditions were found in the MK2 and NK2 treatments, exceeding the CK by 37.68 and 18.77 kg·ha^−1^ mm^−1^, respectively. UHE decreased when irrigation amounts were higher or lower than those in the MK2 and NK2 treatments. This indicates that excessive irrigation is not conducive to improving the UHE of silage maize and instead leads to water waste.

Fertilizer partial productivity (SPFP) is an indicator reflecting the combined effect of local soil basal nutrient levels and fertilizer application rates. Under the same irrigation level, SPFP is inversely proportional to fertilizer application rate (except for reclaimed water levels). Overall, when irrigation reaches reclaimed water levels, the SPFP of each treatment is significantly higher than other treatments. Specifically, in 2023, the SPFP corresponding to the highest yield treatments with and without mulch (MK2, NK2) were 119.19 and 118.98 kg·kg^−1^, respectively, slightly lower than MF1 and NF1 treatments, but higher than the control (CK). In 2024, the SPFP corresponding to the highest yield treatments with and without mulch (MK2, NK2) were 121.62 and 120.18 kg·kg^−1^, respectively, slightly lower than MF1 and NF1 treatments, but higher than the control (CK). Taking into account yield, water use efficiency, and fertilizer partial productivity, and maintaining the target yield within the average yield range, the high-water–fertilizer treatment (135 mm irrigation and 555 kg·ha^−1^ fertilization) is the most suitable irrigation and fertilization combination.

### 2.3. The Water and Fertilizer System Was Optimized in Different Hydrological Years

#### 2.3.1. Characterization of Hydrological Scenarios

The frequency method was used to analyze rainfall data in Tumd Left Banner from 1990 to 2024, a period of 35 years. Based on the results of the frequency method analysis, the years were divided into wet years, normal years, and dry years. A wet year was defined as P ≤ 25%, a normal year as 25% < P < 75%, and a dry year as P ≥ 75%. The calculation formula is as follows:

(1) $$P_{i}=\frac{m}{n+1}\times 100$$

In the formula, P_i_ represents the rainfall frequency in each year, m represents the order of rainfall from maximum to minimum, and n represents the number of years in the data.

**Figure 3:**
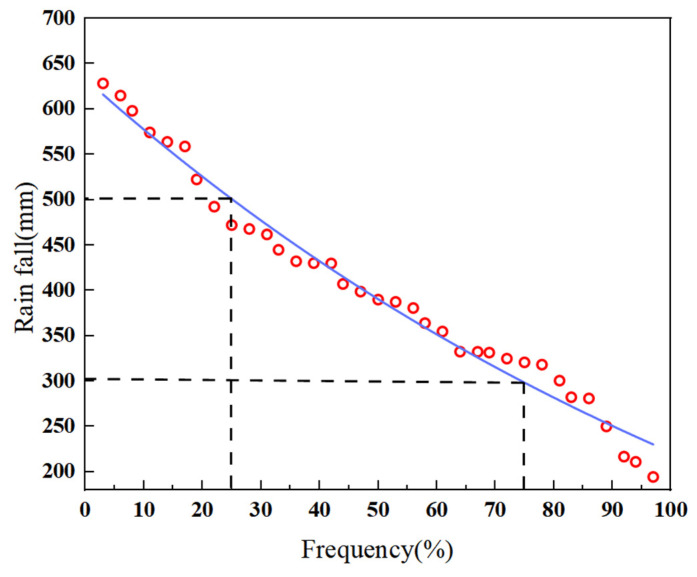
Rainfall frequency curve. The red point represents the historical rainfall data points, and the blue line represents the theoretical frequency distribution curve fitted based on the red point data.

The rainfall frequency curve in Tumote Left Banner is shown in Figure 3. As can be seen from the figure, the rainfall range in wet years is 492.4–628.3 mm, in normal years it is 325–472.2 mm, and in dry years it is 194.6–320.8 mm.

The 35-year period (1990–2024) is divided into 9 dry years, 18 normal years, and 8 abundant years: 1999, 2000, 2005, 2006, 2009, 2011, 2017, 2022, and 2023 are dry years; 1990, 1991, 1992, 1993, 1994, 1995, 1996, 1997, 2001, 2002, 2004, 2007, 2008, 2010, 2014, 2015, 2020, and 2021 are normal years; and 1998, 2003, 2012, 2013, 2016, 2018, 2019, and 2024 are abundant years.

**Figure 4:**
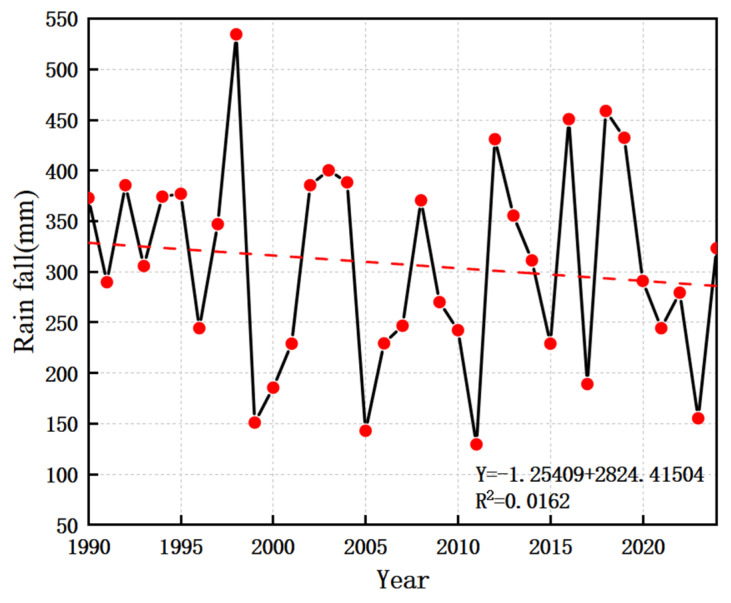
Rainfall during the growth stage of silage maize from 1990 to 2024.

The changes in rainfall during the whole growth period of typical forage crops in Tumut Zuoqi from 1990 to 2024 are shown in Figure 4; the rainfall on silage maize in the growth and development stage shows certain fluctuations, and the difference between the minimum and maximum values is obvious. Silage maize has a large water demand in the growth and development stage, and rainfall has a great impact on its growth and development and the formation of biomass. The average rainfall during the multi-year growth period of silage maize (May–September) was 307.5 mm, the rainfall peaked in 1998 at 534.7 mm, and in 2011 it was underestimated at 129.8 mm, with a maximum value of 4.12 times the minimum. The average rainfall of silage maize in dry years, normal years and wet years was 193 mm, 315 mm and 433 mm, respectively.

The results are shown in Figure 3, and the multi-year rainfall distribution of silage maize shows a slight decreasing trend and a large decreasing trend.

**Figure 5:**
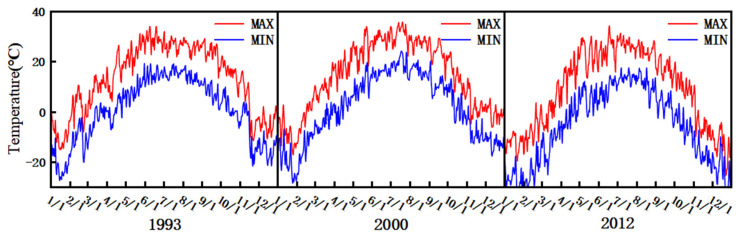
Temperature maps for 1993, 2000, and 2012.

Since the rainfall in 1993, 2000 and 2012 is close to the average rainfall of the corresponding hydrological year, 1993 is selected as a typical normal year, 2000 as a typical dry year, and 2012 as a typical wet year. Figure 5 shows the temperature data maps for 1993, 2000 and 2012. The temperature variation range was −25.7~34.1 °C in 1993, −26.4~35.8 °C in 2000, and −27.2~35.1 °C in 2012. The average temperatures in 1993, 2000 and 2012 were 6.96, 7.96 °C and 7.07 °C, respectively. The average temperatures in the growing seasons in 1993, 2000 and 2012 were 19.86, 21.80 °C and 20.34 °C, respectively, and the average daily maximum temperatures were 34.1, 35.8 and 35.1 °C in June, July and June, respectively.

#### 2.3.2. Synergistic Effects of Water–Fertilizer–Heat Coupling on Aboveground Dry Biomass

**Figure 6:**
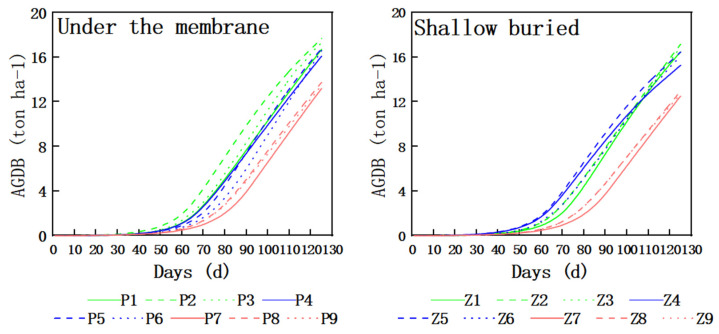
Dry matter mass map of silage corn in different hydrological years. AGDB, aboveground dry biomass.

Figure 6 shows the dynamic changes in aboveground dry matter mass of silage maize with the number of growing days under different hydrological years (wet years, normal years, and dry years). In wet years, drip irrigation under mulch film, with the help of the synergistic effect of “water–fertilizer–heat”, accumulated rapidly as early as 40 days after sowing. P2 (medium water and fertilizer) reached a peak of 17.68 t ha^−1^ at 125 days. However, the aboveground dry matter mass of P3 was low due to high water and fertilizer, indicating that redundant water and fertilizer triggered canopy closure and transpiration stress, and the rate of increase in aboveground dry matter mass dropped sharply. In normal years, the curves of P4–P6 decreased overall, while P5 still maintained a plateau of 16.76 t ha^−1^, indicating that the warming effect of mulch film can partially compensate for the reduction in precipitation. Shallow-buried drip irrigation (Z1–Z9) exhibits a “slow start–steady rise–delayed decline” characteristic. In wet years, although Z2 lags behind by about 15 days in entering linear growth, it maintains a positive slope until 120 days, reaching a final value of 17.16 t ha^−1^. The peak value of Z5 decreases slightly while the decline is slow, but it still reaches 16.46 t ha^−1^, the highest in normal years. In dry years, the difference between P7–P9 and Z7–Z9 is the most significant, with a maximum difference of 0.78 t ha^−1^. P8 and Z8 reach the highest values in subsurface drip irrigation and shallow-buried drip irrigation, at 13.73 and 12.96 t ha^−1^, respectively.

#### 2.3.3. Trade-Offs Between Yield, Water Use Efficiency and Partial Fertilizer Productivity for Regime Optimization

Based on the classification of different hydrological years from 1990 to 2024 and the statistical analysis of rainfall during the growth period of silage maize, the specific rainfall amounts for each growth stage of silage maize under different hydrological years were clarified. The rainfall values for the growth stages of silage maize in dry years, normal years, and wet years were 193 mm, 315 mm, and 433 mm, respectively. Through the analysis of different hydrological years, 2000 was selected as a typical dry year, 1993 as a typical normal year, and 2012 as a typical wet year.

As shown in Table 6, drip irrigation under mulch promotes rapid growth in the early growth stage through a significant warming effect. Under treatment P2 (water–fertilized water) in a wet year, the highest yield of 70.4 t ha^−1^ was achieved, with a water use efficiency of 135.1 kg ha^−1^ mm^−1^. However, excessive water and fertilizer (P3) reduced the fertilizer partial productivity to 126.9 kg kg^−1^, indicating that the coupling of “water–fertilizer–heat” under mulch conditions is prone to triggering physiological redundancy. In contrast, the shallow-buried drip irrigation treatment Z2 (water–fertilized water) yielded 68.5 t ha^−1^, with a water use efficiency of 131.5 kg ha^−1^ mm^−1^ and a fertilizer partial productivity of 123.4 kg kg^−1^. In normal water years, the P5 (medium water fertilization) drip irrigation treatment under mulch film had the highest water use efficiency (152.2 kg ha^−1^ mm^−1^), while the Z5 (medium water fertilization) shallow-buried drip irrigation treatment maintained high yield and efficiency with a water use efficiency of 149.2 kg ha^−1^ mm^−1^ and a fertilizer partial productivity of 118.6 kg kg^−1^. In dry years, the yield of both irrigation methods decreased to 45.1–48.9 t ha^−1^, but the Z7 (low water fertilization) shallow-buried drip irrigation treatment had a water use efficiency of 123.3 kg ha^−1^ mm^−1^ and a fertilizer partial productivity of 101.6 kg kg^−1^, significantly lower than the P7 (low water, high fertilizer) drip irrigation treatment under mulch film (127.9 kg ha^−1^ mm^−1^ and 105.4 kg kg^−1^). This indicates that shallow-buried drip irrigation achieves “yield reduction without efficiency decrease” under extreme drought conditions by reducing inter-plant evaporation and improving root zone water use efficiency. In summary, drip irrigation under mulch is suitable for maximizing yields in years with abundant water, while shallow-buried drip irrigation achieves a three-dimensional synergy of “yield–water–fertilizer” through water conservation and fertilizer control in years with normal and low water levels.

## 3. Discussion

Based on field measurement data from 2023 to 2024, this study calibrated and validated the AquaCrop model for silage maize growth simulation under sandy soil and mulching/shallow drip irrigation conditions in the Yellow River irrigation area of Inner Mongolia. The results showed that the model met the simulation requirements for soil moisture content (RMSE < 3.3%, EF > 0.7, 0.85 ≤ R^2^ ≤ 0.95), canopy coverage (RMSE < 3.95%, EF > 0.79, 0.85 ≤ R^2^ ≤ 0.94), and aboveground biomass (RMSE < 4.5 t·ha^−1^, EF > 0.75, 0.85 ≤ R^2^ ≤ 0.94), and was basically consistent with the results of domestic and foreign studies [28,29,30]. This confirms that the model can serve as a reliable basis for optimizing the water and fertilizer system for silage maize in this region.

(1) Water and fertilizer utilization strategies for crops under drip irrigation under film to cope with combined water, fertilizer and heat stress.

Drip irrigation under film regulates the allocation of “water–fertilizer–heat” resources through the core effect of film heating and moisture retention. Under the combined stress of water, fertilizer and heat, it achieves high yield and efficient resource synergy of silage corn. Its stress relief mechanism is manifested in the fact that film covering increases the soil temperature of the cultivated layer and reduces evaporation between plants, effectively alleviating low temperature and water stress, creating a suitable environment for fertilizer absorption, and significantly promoting the rapid accumulation of dry matter in the early growth stage (the effective accumulated temperature of 900 °C·d enters the linear growth period), which is consistent with the law of film heating and moisture retention to promote early growth proposed by Qi Yinglong et al. [31]. In terms of the optimal water and fertilizer threshold, medium fertilizer (555 kg·ha^−1^) is the stable optimal fertilizer amount across hydrological years. Excessive fertilizer application is prone to cause canopy redundancy growth, resulting in a decrease in fertilizer productivity. The irrigation amount needs to be dynamically adjusted with the hydrological year (90 mm in wet years, 135 mm in normal years, and 180 mm in dry years), which can balance water and heat stress, which is consistent with the conclusion of Zhao et al. [32] that a medium amount of water and fertilizer achieves efficient resource coupling. In terms of the hydrological year adaptation effect, in wet years, relying on precipitation for supplementation, the high yield, high efficiency and superior quality of the medium water fertilization treatment (yield 70.4 t·ha^−1^, WUE 135.1 kg·ha^−1^·mm^−1^) are consistent with the pattern of no irrigation + 240 kg·ha^−1^ nitrogen application in wet years in Henan Province, reaching 98~100% of the potential yield. Moreover, the fertilizer partial productivity caused by excessive water and fertilizer is reduced to 126.9 kg·kg^−1^, which is consistent with the significant reduction in PFPN after nitrogen application exceeding 240 kg·ha^−1^ in this region [33]. In dry years, it is necessary to increase irrigation to 180 mm to compensate for insufficient precipitation and alleviate the superposition of multiple stresses. This phenomenon is consistent with the irrigation demand characteristics of the mulched area in extremely dry years of the Huang–Huai–Hai Plain [34].

(2) Water and fertilizer utilization strategies for crops under shallow-buried drip irrigation to cope with combined water, fertilizer and heat stress

Shallow-buried drip irrigation reduces inter-plant evaporation through capillary burial depth, and exhibits the advantages of “water saving and stable efficiency” under combined water, fertilizer and heat stress. Its stress relief mechanism is that the capillary burial depth of 5 cm reduces the ineffective consumption of surface water and relieves water stress. Although the lack of mulching warming effect leads to early growth lag, the water and fertilizer supply is stable in the middle and late stages, which can avoid the risk of late water deficit and high-temperature stress superimposed. This is consistent with the local water control mode of Liu et al. [35], to reduce consumption and stabilize yield, and the mechanism of stable supply and efficiency enhancement in the late stage of non-mulching covering proposed by Mei Siwei et al. [36]. Shallow-buried drip irrigation has a slow early growth and a 2.7% lower yield due to the lack of mulching warming, which is consistent with the observation results of insufficient soil accumulated temperature and slowed crop growth rate in the early stage when shallow-buried drip irrigation is not covered in the Xiliao River Basin [37]. The optimal water and fertilizer threshold is consistent with that of drip irrigation under film. Medium fertilizer (555 kg·ha^−1^) can prevent fertilizer stress. The irrigation amount is adjusted according to the hydrological year to 90 mm in wet years, 135 mm in normal years, and 180 mm in dry years, so as to maximize water use efficiency while ensuring yield. In terms of the hydrological year adaptation effect, the advantages of normal years and dry years are significant. In dry years, the medium-water and -fertilizer treatment (180 mm, 555 kg ha^−1^) yields 46.8 t·ha^−1^ and water use efficiency is 127.94 kg·ha^−1^·mm^−1^, achieving “limited production without reduced efficiency”, which corresponds to the irrigation pattern of dry years in eastern Inner Mongolia [38]. In wet years, the yield is slightly lower than that of drip irrigation under film due to the lack of warming effect, but the water-saving advantage is obvious. Compared with drip irrigation under film, the water use efficiency and fertilizer partial productivity of shallow-buried drip irrigation decreased by 1.52–5.42% and 1.53–5.42%, respectively, under the same water and fertilizer configuration. However, it has stronger stress resistance under water-shortage scenarios and is more suitable for the sustainable production needs of water-scarce areas. The yield difference between the two drip irrigation modes is the largest in dry years (4.1%) and the smallest in wet years (2.7%), highlighting the risk resistance advantage of drip irrigation under water-shortage scenarios [39].

Silage maize shows significant physiological plasticity to extreme water conditions: under drought conditions, it maintains cell turgor pressure through stomatal regulation and root architecture adjustment [40]; in the case of excess water, hypoxia and nutrient leaching are alleviated by regulating root zone aeration [41]. The dynamic irrigation threshold (from 90 mm in wet years to 180 mm in dry years) simulated in this study utilized these biological mechanisms: in dry years, 180 mm supplemental irrigation supported the osmotic regulation of crops and prevented leaf senescence; in wet years, the irrigation amount reduced to 90 mm optimized the fertilizer–water–air ratio of high-permeability sand and avoided the ‘physiological drought’ caused by hypoxia and nutrient leaching in the root zone. By combining with the inherent adaptation strategy of crops, the ‘hydrological year-based’ management scheme proposed in this study effectively balances the relationship between crop survival mechanism and yield maximization under multiple abiotic stresses.

While this study clarified the optimal water and fertilizer regime for silage maize under different drip irrigation modes and hydrological year types, it still has three limitations: First, the static water and fertilizer regime design did not consider the dynamic differences in water and fertilizer requirements at different growth stages of silage maize, which may lead to a mismatch between water and fertilizer supply and demand during key growth periods. Second, the scenario simulation did not include long-term climate change factors (such as increased CO_2_ concentration and extreme high-temperature events), making it difficult to reflect the adaptability of the water and fertilizer regime under future climate scenarios. Therefore, future research should focus on the construction of dynamic water and fertilizer models and the analysis of multi-climate scenario simulations to improve the precision water and fertilizer management system for silage maize in arid areas.

## 4. Materials and Methods

### 4.1. Overview of the Study Area

**Figure 7:**
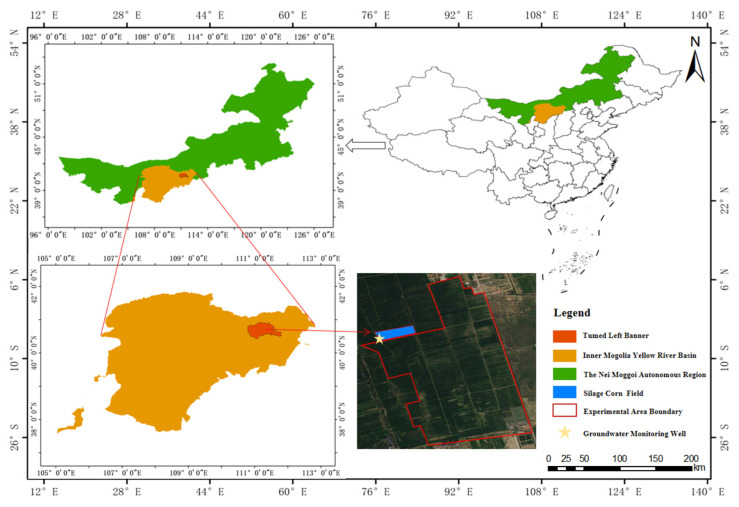
Study area overview map.

The Yellow River Basin in Inner Mongolia is located at the northernmost end of the Yellow River (37°35′−41°50′ N, 106°10′−112°50′ E). Its total usable conventional water resources amount to 5.145 billion m^3^, with a per capita usable water resource of 856 m^3^, resulting in a rigid water shortage of 1.816 billion m^3^. Its cultivated land area is 3.08 million hectares, accounting for 27.31% of the region’s total cultivated land area. Annual precipitation is 250–300 mm. Major crops include maize, silage maize, wheat, oats, and alfalfa. The study area is located in Tumd Left Banner, Hohhot City (40°38′−40°44′ N, 111°16′−111°24′ E), with a gentle terrain and a continental monsoon climate. The study area covers a total area of 170.67 hectares, with 0.51 hectares planted with silage corn. The average annual precipitation is 400 mm, concentrated in the summer months of June to August. The average daily maximum temperature is 28.7 °C, occurring in June and July, while the average daily minimum temperature is 11.3 °C, occurring in May. The average annual evaporation is 1870.3 mm, the average annual sunshine duration is 2876.5 h, and the annual total solar radiation is 133.82 kcal/cm^2^. The frost-free period is relatively short (approximately 133 days). The soil in the study area is predominantly sandy loam (0–40 cm) and sandy below 40 cm. Drip irrigation is used for silage corn cultivation.The regional overview is shown in Figure 7.

### 4.2. Experimental Design

**Figure 8:**
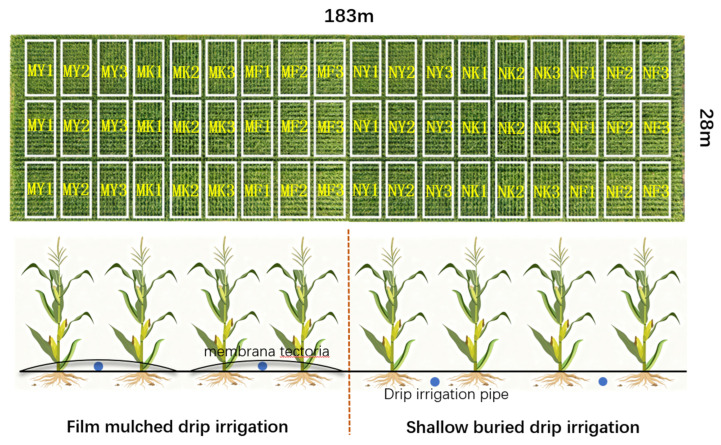
Experimental layout diagram. Coating type, PBAT-based biodegradable mulch; blue dot, drip irrigation pipe.

In this study, field experiments were carried out in two consecutive growing seasons (May to September 2023 and 2024) in the demonstration area of Tumd Left Banner, Hohhot, Inner Mongolia. The data in 2023 was used for model calibration, and an independent dataset was provided for model verification in 2024. The irrigation amount was determined according to the local irrigation quota (180 mm) and set as a 100% benchmark. Three irrigation levels were set: low water (50% of the local amount, i.e., 90 mm), medium water (75%, 135 mm) and high water (100%, 180 mm). Similarly, the fertilization level was designed according to the gradient of local traditional fertilization (total 1125 kg ha^−1^). The treatments included low fertilizer (300 kg ha^−1^ base fertilizer, 72 kg ha^−1^ topdressing), medium fertilizer (375 kg ha^−1^ base fertilizer, 90 kg ha^−1^ topdressing) and high fertilizer (450 kg ha^−1^ base fertilizer, 108 kg ha^−1^ topdressing). The base compound fertilizer contains 18% N, 22% P_2_O_5_ and 5% K_2_O, and the topdressing urea contains 46.8% N. The control group (CK) followed a complete local standard (irrigation 180 mm, fertilization 1125 kg ha^−1^). No trace elements were added in this experiment. Each treatment is shown in Table 7 and Table 8. The area was divided into 54 test cells (9 × 10 m) and equipped with protective belts. The variety of silage corn was DK808, the growth period was May–September, the irrigation method was drip irrigation under the film and shallow-buried drip irrigation (buried depth 5 cm), the planting area was 0.51 hectares, the drip irrigation belt spacing was 45 cm, the plant spacing was 25 cm, and each drip irrigation belt controlled 2 rows of silage corn. The test layout diagram is shown in Figure 8. According to the actual situation, there is no runoff in the field and the weed coverage rate is 5%.

The model was calibrated using the measured values in 2023 and verified using the measured data in 2024. The soil moisture content, canopy coverage and aboveground biomass (yield) of the model were calibrated and verified by the trial-and-error method. The soil moisture and canopy coverage were sampled and tested using a five-point sampling method, with each point repeated three times and measured once every 7 days (The measured values of soil moisture content are shown in Tables S3 and S4). Similarly, the aboveground biomass (yield) was also sampled and tested using a five-point sampling method, with each point repeated three times and measured once every 15 days (The measured values are shown in Table S1 and S2). Among them, for aboveground biomass (yield), 5 plants that could represent the average growth were taken from each plot, and then the aboveground parts were cut off, blanched at 105 °C for 0.5 h, dried at 75 °C to constant weight, and the dry weight was measured. The aboveground biomass (yield) was obtained by multiplying the dry weight by the planting density. Soil moisture content was determined by the drying method. Crop height was measured with a tape measure. Canopy coverage was calculated using the leaf area index, with an extinction coefficient of 0.7 [42]. Before the experiment, soil texture at each monitoring point was determined using the Wilkes soil particle size distribution method (Table 9). Sampling depths were 0–10 cm, 10–20 cm, 20–40 cm, 40–60 cm, and 60–80 cm. One groundwater level monitoring well was located within the experimental field (monitored every 7 days, Figure 2). Daily meteorological data for rainfall, radiation, air temperature, wind speed, and humidity were obtained from the automatic weather station in the experimental area (HOBO-U30, Onset Computer Corp., Bourne, MA, USA).

The irrigation water source in this study area is local groundwater, which does not carry the risk of soil salinization. The drip irrigation pipe used in the experiment was replaced once a year, and the frequency of field irrigation was low. At the same time, the microbial content of groundwater in the study area was very low, and all fertilization treatments were applied with water-soluble fertilizer, which effectively eliminated the risk of emitter blockage. In addition, under the experimental conditions, there was no potential risk of salinization and alkalization in the soil of the study area.

### 4.3. Basic Principles of the Model

The AquaCrop model is a general-purpose crop growth simulation model developed by the Food and Agriculture Organization of the United Nations (FAO) to assess the impact of food security and environmental management on crop production. The model achieves a good balance between simplicity, accuracy and stability of simulation with fewer parameters, and is widely used in deficit irrigation designation strategies [43,44,45]:

The theoretical basis is based on the following yield moisture response relationship:

(2) $$\left(1-\frac{Y}{Y_{x}}\right)=K_{y}\left(1-\frac{ET}{{ET}_{x}}\right)$$

where Y_x_ and Y represent the potential yield and actual yield of crops, respectively, kg·ha^−2^; K_y_ represents the scale factor of crop yield response to moisture; ET_x_ and ET represent the potential transpiration and actual transpiration of the crop, mm.

After evolution and improvement, the formula for calculating crop transpiration is as follows:

(3) $$T_{r}=K_{s}K_{s_{Tr}}\left(K_{c_{Tr,x}}{CC}^{*}\right){ET}_{o}$$

The formula for calculating soil evaporation in this model is

(4) $$E_{s}=K_{r}\left(1-{CC}^{*}\right){Ke}_{x}{ET}_{o}$$

In the formula, T_r_ represents crop transpiration (mm); K_s_ represents the soil moisture stress coefficient (%); K_sTr_ represents the temperature stress coefficient; K_CTr,x_ represents all the different factors that distinguish the actual crop from the reference crop (%); CC* represents the calibrated canopy cover (%); ET_o_ represents crop evapotranspiration (mm day^−1^); K_e,x_ is the maximum soil evaporation coefficient; and Kr is the evaporation reduction coefficient (ranging from 0 to 1). When the surface soil moisture is insufficient to meet the atmospheric evaporation demand, K_r_ < 1.

Secondly, to measure the stress effects of soil moisture and temperature on maize growth, stress functions for soil moisture and soil temperature were introduced respectively and integrated into the decision-making model for saline–freshwater alternating drip irrigation farmland. The functional relationship is as follows:

Water stress formula:

(5) $$K_{S}=\left\{\begin{array}{c} \begin{array}{cc} 1 & \theta >\theta_{fc} \end{array}\\ \begin{array}{cc} \frac{\theta -\theta_{wp}}{\theta_{fc}-\theta_{wp}} & \theta_{wp}<\theta \leq \theta_{fc} \end{array}\\ \begin{array}{cc} 0 & \theta \leq \theta_{wp} \end{array} \end{array}\right.$$

Temperature stress formula:

(6) $$K_{sTr}=\left\{\begin{array}{l} \begin{array}{cc} \;0 & \;T_{s}\leq 0 \end{array}\\ \begin{array}{cc} 1-1.6\times 10^{-3}{\left(25-T_{s}\right)}^{2} & 0<T_{s}<25 \end{array}\\ \begin{array}{cc} \;1 & \;T_{s}\geq 25 \end{array} \end{array}\right.$$

In the formula, θ_wp_ represents the wilting coefficient (cm^3^ cm^−3^), θ_fc_ represents the field water-holding capacity (cm^3^ cm^−3^), and Ts represents the soil temperature (°C).

The formula for calculating aboveground biomass is

(7) $$B={WP}^{*}\sum_{i}\frac{{Tr}_{i}}{{ETo}_{i}}$$

B represents biomass, t·ha^−2^; T_ri_ and ET_oi_ are the crop transpiration (mm day^−1^) and reference crop evapotranspiration (mm day^−1^) on day i, respectively.

When CC ≤ CC_x_/2, the canopy coverage increases exponentially, and the calculation formula is

(8) $$CC={CC}_{o}e^{tCGC}$$

When CC > CC_x_/2, the canopy coverage index attenuates as follows:

(9) $$CC={CC}_{x}-0.25\frac{{\left({CC}_{x}\right)}^{2}}{{CC}_{o}}e^{-tCGC}$$

CC represents canopy coverage at time t; CC_o_ represents the initial canopy size; CC_x_ represents maximum canopy coverage; CGC stands for canopy growth coefficient; t stands for time, d.

The AquaCrop model does not explicitly consider nutrient cycling and balance, but uses a semi-quantitative method to describe the impact of nutrient stress on crop growth, with the following formula:

(10) $$B_{rel}=\frac{B_{stress}}{B_{ref}}\times 100\%$$

In the formula, B_stress_ is the aboveground biomass (kg·ha^−1^) obtained under fertilizer stress; B_ref_ is the aboveground biomass (kg·ha^−1^) obtained without fertilizer stress; Brel ranges from 0 to 100%; and B_stress_ and B_ref_ are determined from the data of this experiment. Five stress parameters are used to reflect the response to fertilizer stress: relative biomass, maximum canopy cover under fertilizer stress (CC_xstress_), canopy attenuation degree, average canopy reduction, and canopy expansion reduction.

### 4.4. Data Acquisition

#### 4.4.1. Meteorological Data

**Figure 9:**
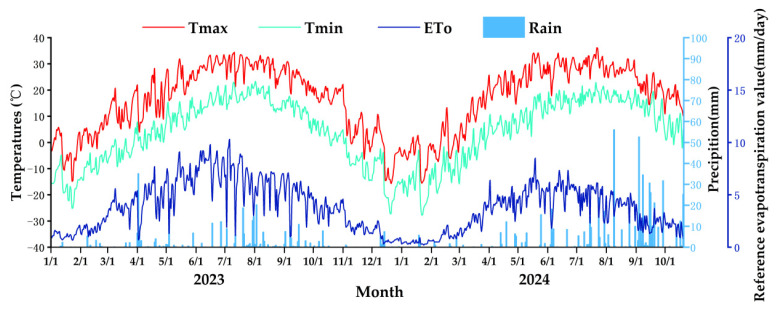
Meteorological maps for 2023 and 2024. T_max_, maximum temperature; T_min_, minimum temperature; ET_0_, evapotranspiration.

The meteorological data required for this study were obtained from automatic weather stations located in the experimental area (HOBO-U30, Onset Computer Corp., Bourne, MA, USA). Figure 2 shows the temperature and precipitation statistics for 2023 and 2024. The annual precipitation in 2023 was 320.81 mm, and the annual precipitation in 2024 was 598.10 mm. The temperature range in 2023 was −27 to 34.41 °C, and the temperature range in 2024 was −27.8 to 36 °C. The average temperatures in 2023 and 2024 were −21.5 to 28.4 °C and −21.8 to 28.7 °C, respectively. Rainfall during the growing season (5 May–6 September 2023, and 5 May–6 September 2024) was 155.5 mm and 323.5 mm, respectively, accounting for 48.47% and 54.09% of the annual rainfall. The average temperatures during the growing season in 2023 and 2024 were 21.75 °C and 22.14 °C, respectively. The average daily maximum temperatures occurred in July, at 28.04 °C and 28.7 °C, respectively. Crop evapotranspiration (ET_o_) was calculated using the Penman–Monteith model and the Monteith formula. The meteorological maps for 2023 and 2024 are shown in Figure 9.

#### 4.4.2. Soil Data

Five layers were sampled at the experimental site, with sampling depths of 0–10 cm, 10–20 cm, 20–40 cm, 40–60 cm, and 60–80 cm. Three samples were collected from each layer. The undisturbed soil samples were soaked to saturation using the Wilcox method [46] and then placed on the air-dried soil. The gravitational water in the soil samples was drained by suction of the air-dried soil. After a period of time, the field water-holding capacity, saturated water content, and soil bulk density of the soil were determined, and the soil particle size distribution at each point was determined using a dry particle size analyzer. The sand content, silt content, and clay content in each soil layer are shown in Table 9.

#### 4.4.3. Crop Growth Period

Silage corn is planted in early May. In 2023, the planting date was 5 May, and the harvest date was 6 September. In 2024, the planting date was 6 May, and the harvest date was 7 September. The growing period for both was 125 days, with the rapid growth period occurring around June or July, lasting up to 27 days.The growth period of silage corn is shown in Table 10.

**Table 10:** Statistics on the growth period of silage corn in 2023 and 2024.

Year	Crops	Planting Time	Harvest Time	Growth Stage				Total Days (d)
				Seedling	Propagation	Ecchymosis	Maturity	
2023	Silage Corn	5 May	6 September	39	26	32	28	125
2024	Silage Corn	6 May	7 September	37	28	31	29	125

#### 4.4.4. Groundwater Depth

**Figure 10:**
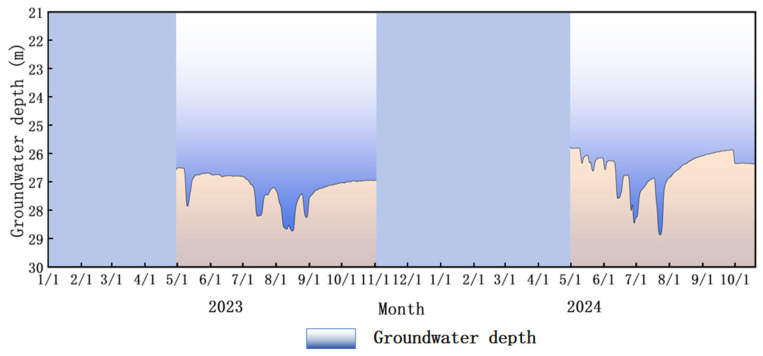
Groundwater depth map.

The experimental site is dominated by deep groundwater, with an average annual depth of approximately 26 m. The average groundwater depth during the growing season in 2023 was 27.3 m, and in 2024 it was 26.7 m. June, July, and August represent peak irrigation periods, during which the groundwater level fluctuated significantly. After crop harvest, water consumption decreased, leading to a rise in the groundwater level. With the implementation of water-saving measures in recent years and the relatively high rainfall in 2024, the groundwater depth has gradually increased. However, considering that the groundwater depth exceeds 3 m, its impact on this study is negligible. The groundwater depth map is shown in Figure 10.

#### 4.4.5. Crop Parameters

The crop parameters include crop development stage, evapotranspiration, crop yield, and soil moisture stress, soil fertility stress, and temperature stress. Crop growth parameters CC_x_, number of plants per hectare, CC_o_, and Z_max_ are obtained through field observations. Baseline temperature and upper temperature limit use the C4 crop parameter values recommended by the model. Harvest index (HI_0_), canopy growth coefficient (CGC), canopy decay coefficient (CDC), standard water productivity (WP*), crop coefficient (K_cTr, x_) with intact but senescent canopy, water stress parameters (P_exp, upper_, P_exp, lower_, P_sto, upper_, P_sen, upper_), and fertility stress parameters are determined based on the reference parameters provided by the model, and are corrected using a trial-and-error method.

### 4.5. Simulation of Different Water and Fertilizer Scenarios

To explore the effects of different water and fertilizer regimes, such as drip irrigation under mulch and shallow-buried drip irrigation, on the yield and water productivity of silage maize, this paper used meteorological data from 1990 to 2024 for a total of 35 years as a basis, and based on field experiments in 2023–2024 and local water and fertilizer regimes, different simulation scenarios were developed for different hydrological years and under water-limited conditions, as shown in Table 11 and Table 12.

### 4.6. Parameter Calculation

The effective accumulated temperature refers to the sum of the effective temperatures of organisms during a certain or all growth periods, and is calculated as follows [47]:

(11) $$T_{i}=\sum_{i=1}^{n}\frac{T_{max}-T_{min}}{2}-T_{base}$$

In the formula, T_i_ is the effective accumulated temperature (°C); n is the growing season; T_max_ and T_min_ are the highest and lowest temperatures of the day (°C); T_base_ is the minimum temperature required for crop physiological activities, which is 10 °C for silage corn.

Water productivity refers to the dry matter produced by the evaporation of a unit mass of water consumed by crops in the field. The unit is kg m^−3^. It reflects the energy conversion efficiency in the plant production process. It is an indicator for measuring the relationship between crop yield and water consumption. It is also one of the comprehensive indicators for evaluating the suitability of plant growth under water deficit. The calculation formula is as follows [48]:

(12) $$WP=\frac{Y}{ET}$$

In the formula, WP is the water productivity (kg m^−3^); Y is the yield (kg da^−1^); and ET is the plant evapotranspiration (mm).

### 4.7. Calibration and Validation

This study used soil moisture content, canopy coverage index and yield in 2023 for parameter calibration, and used data from 2024 to verify the simulation accuracy of the model. The model calibration and verification were performed using root mean square error (RMSE), coefficient of determination (R^2^) and model performance index (EF) [20]:

Root mean square error:

(13) $$RMSE=\sqrt{\frac{\sum_{i=1}^{n}{\left(M_{i}-S_{i}\right)}^{2}}{n}}$$

Model performance index:

(14) $$EF=\frac{\sum_{i=1}^{n}(M_{i}{-\bar{M})}^{2}-\sum_{i=1}^{n}{\left(M_{i}-S_{i}\right)}^{2}}{\sum_{i=1}^{n}{\left(M_{i}-\bar{M}\right)}^{2}}$$

Coefficient of determination:

(15) $$R^{2}={\left[\frac{\sum_{i=1}^{n}\left(M_{i}-\bar{M})(S_{i}-\bar{S}\right)}{{\left[\sum_{i=1}^{n}{\left(M_{i}-\bar{M}\right)}^{2}\right]}^{0.5}{\left[\sum_{i=1}^{n}{\left(S_{i}-\bar{S}\right)}^{2}\right]}^{0.5}}\right]}^{2}$$

In the formula, n is the number of samples; M_i_ and S_i_ are the measured and simulated values of the parameters, respectively; M is the measured average value. RMSE is used to describe the magnitude of the error in model estimation, and the smaller the value, the better; EF is used to describe the relative error of the model, and its value is between 0 and 1. The closer it is to 1, the smaller the deviation between the simulated value and the measured value, that is, the better the model simulation effect.

## 5. Conclusions

This study calibrated and validated the AquaCrop model through field trials in 2023 and 2024. It systematically analyzed the regulatory effects of shallow-buried drip irrigation and mulching drip irrigation on the productivity and resource utilization efficiency of silage maize from multiple dimensions, including drip irrigation mode, water–fertilizer coupling, and hydrological year patterns. The following conclusions were drawn:

(1) The simulation values of the AquaCrop model have high fitting accuracy with the observed data, indicating that it has high reliability in simulating the growth of silage corn.

(2) The dynamic trends of canopy coverage of silage maize over the two years were similar, indicating that the model’s ability to simulate this indicator was stable in different years. Subsurface drip irrigation triggered rapid accumulation of dry matter at 900 °C·d, with a peak of 66.7 t ha^−1^. Shallow-buried drip irrigation, due to reduced evaporation at burial depth, maintained a maximum rate of approximately 73.5 kg ha^−1^ °C^−1^ at 1000~1230 °C·d. Water–fertilizer (135 mm, 555 kg ha^−1^) is a common “high-yield–high-efficiency” threshold for both drip irrigation methods; beyond this threshold, the marginal benefits of water and fertilizer decrease significantly.

(3) The adaptability of drip irrigation modes to hydrological year patterns varies significantly. Subsurface drip irrigation relies on the ecological niche advantage of the mulch film in warming and moisture retention. In wet years, (90 mm, 555 kg ha^−1^) is preferred, while (135 mm, 555 kg ha^−1^) and (180 mm, 555 kg ha^−1^) are suitable for normal and dry years, respectively. Shallow-buried drip irrigation also shows the synergistic advantage of “water saving and high efficiency”. In wet years, (90 mm, 555 kg ha^−1^) is preferred, while (135 mm, 555 kg ha^−1^) and (180 mm, 555 kg ha^−1^) are suitable for normal and dry years, respectively. Under the same water and fertilizer configuration, the water use efficiency and fertilizer partial productivity of shallow-buried drip irrigation are reduced by 1.52~5.42% and 1.53~5.42%, respectively, compared with subsurface drip irrigation.

The ‘drip irrigation mode–water and fertilizer level–hydrological year type’ adaptation system constructed in this study provides a robust quantitative basis for water and fertilizer management for pre-growth-period planning and mid-growth-period scheduling of silage maize in arid and semi-arid irrigation areas. Although the AquaCrop model shows extremely high efficiency in formulating water-saving strategies due to its water-driven engine and low parameter requirements, it is limited to static water and fertilizer system optimization and lacks refined dynamic judgment and system optimization. According to the needs of relevant policies, this experiment will improve the optimization of water and fertilizer systems in dynamic changes by combining the model with the Internet of Things (IoT) and remote sensing in the future, and provide a basis for the implementation of relevant policies.

## Figures and Tables

**Table 1:** AquaCrop model calibration evaluation.

Year	Irrigation Method	Treatment	Soil Moisture Content			Canopy Coverage		Aboveground Biomass (Yield)			
			RMSE/%	EF	R^2^	RMSE/%	EF	R^2^	RMSE/ton ha^−1^	EF	R^2^
2023 (model calibration)	Drip irrigation under mulch	MY1	2.54	0.74	0.85	3.92	0.91	0.94	3.1	0.92	0.94
		MY2	3.18	0.75	0.89	3.26	0.86	0.94	4.0	0.85	0.92
		MY3	3.09	0.73	0.86	3.49	0.84	0.89	4.3	0.83	0.86
		MK1	2.59	0.85	0.86	2.98	0.85	0.91	4.1	0.79	0.89
		MK2	3.25	0.80	0.87	3.04	0.85	0.90	3.8	0.90	0.85
		MK3	2.43	0.73	0.87	2.89	0.93	0.87	3.1	0.85	0.91
		MF1	2.84	0.86	0.87	3.15	0.91	0.87	4.0	0.81	0.91
		MF2	2.82	0.74	0.87	3.72	0.80	0.89	3.5	0.92	0.87
		NF3	2.64	0.78	0.88	3.56	0.85	0.88	3.7	0.96	0.89
		CK	2.54	0.74	0.85	3.53	0.82	0.86	3.9	0.94	0.86
	Shallow-buried drip irrigation	NY1	2.54	0.78	0.86	3.51	0.83	0.91	3.1	0.84	0.94
		NY2	2.49	0.90	0.87	3.84	0.86	0.93	4.0	0.81	0.92
		NY3	2.85	0.83	0.88	2.97	0.81	0.89	4.3	0.86	0.88
		NK1	2.75	0.76	0.86	3.05	0.79	0.85	4.1	0.87	0.88
		NK2	3.07	0.73	0.86	3.42	0.87	0.87	3.8	0.90	0.86
		NK3	2.64	0.74	0.85	3.61	0.93	0.88	3.1	0.85	0.89
		NF1	2.53	0.84	0.86	3.28	0.86	0.90	4.0	0.86	0.91
		NF2	3.17	0.71	0.87	2.86	0.91	0.92	3.5	0.91	0.87
		NF3	3.17	0.71	0.87	2.97	0.89	0.90	3.2	0.85	0.90
		CK	2.46	0.92	0.87	3.66	0.94	0.89	3.9	0.93	0.86

**Table 2:** AquaCrop model silage corn parameter list.

Parameter	Description	Taking Values	Unit
HI_0_	Reference Harvest Index	45	
CGC	Canopy growth coefficient	9.6	%/d
CDC	Canopy attenuation coefficient	6	%/d
CC_x_	Maximum canopy coverage	95	%
WP *	Standard water productivity	35	g/m^2^
T_base_	Base temperature	5	°C
T_upper_	Upper limit temperature	35	°C
K_c_T_r,x_	Crop coefficient when canopy is complete but prior to senescence	1.30	-
CC_o_	Initial canopy coverage	0.32	%
P_exp,upper_	Upper limit of effect of water stress on canopy	0.14	
P_exp,lower_	Lower limit of influence of water stress on canopy	0.72	
P_sto,upper_	Upper limit of water stress on stomatal conductance	0.30	
P_sen,upper_	Upper limit of effect of water stress on early canopy senescence	0.80	
Z_max_	Maximum effective root depth	0.8	m
Z_min_	Minimum effective root depth	0.1	m
	Number of plants per hectare	110,000	Plants/ha^2^

**Table 3:** Fertilizer stress parameter values in the AquaCrop model.

Fertilization Level	Parameter	Taking Values	Unit
High fertilizer	Relative biomass	88	%
	CC_xstress_	82	%
	Relative biomass canopy decay	Small	
	Average canopy reduction	0.01	%
	Reduced canopy expansion	3	%
Medium fertilizer	Relative biomass	60	%
	CC_xstress_	77	%
	Relative biomass canopy decay	Small	
	Average canopy reduction	0.01	%
	Reduced canopy expansion	5	%
Low fertilizer	Relative biomass	55	%
	CC_xstress_	67	%
	Relative biomass canopy decay	Small	
	Average canopy reduction	0.03	%
	Reduced canopy expansion	13	%

**Table 4:** AquaCrop model validation evaluation.

Year	Irrigation Method	Treatment	Soil Moisture Content			Canopy Coverage			Aboveground Biomass (Yield)		
			RMSE/%	EF	R^2^	RMSE/%	EF	R^2^	RMSE/ton ha^−1^	EF	R^2^
2024 (model verification)	Drip irrigation under mulch	MY1	2.49	0.82	0.86	3.28	0.79	0.86	3.8	0.81	0.89
		MY2	2.65	0.83	0.91	3.45	0.83	0.89	3.9	0.79	0.87
		MY3	2.58	0.87	0.89	3.39	0.81	0.91	3.5	0.83	0.92
		MK1	2.61	0.81	0.87	3.52	0.83	0.90	3.3	0.75	0.88
		MK2	2.53	0.85	0.88	3.67	0.80	0.87	3.7	0.84	0.88
		MK3	2.63	0.85	0.89	3.65	0.84	0.85	3.5	0.81	0.91
		MF1	2.51	0.86	0.86	3.19	0.85	0.86	3.9	0.76	0.89
		MF2	2.58	0.84	0.92	3.24	0.82	0.87	3.3	0.82	0.90
		NF3	2.54	0.81	0.90	3.39	0.81	0.93	3.4	0.81	0.86
		CK	2.64	0.82	0.91	3.46	0.86	0.89	3.8	0.80	0.87
	Shallow-buried drip irrigation	NY1	2.51	0.82	0.90	3.25	0.87	0.91	3.3	0.78	0.89
		NY2	2.62	0.81	0.91	3.14	0.85	0.88	3.7	0.79	0.91
		NY3	2.55	0.86	0.87	3.56	0.88	0.86	3.9	0.83	0.86
		NK1	2.48	0.83	0.89	3.47	0.87	0.86	3.6	0.81	0.89
		NK2	2.61	0.83	0.88	3.69	0.89	0.89	3.4	0.85	0.88
		NK3	2.54	0.84	0.87	3.71	0.84	0.91	3.5	0.86	0.87
		NF1	2.51	0.86	0.91	3.75	0.88	0.92	3.7	0.83	0.93
		NF2	2.57	0.84	0.93	3.64	0.84	0.90	3.2	0.80	0.93
		NF3	2.63	0.85	0.94	3.52	0.89	0.89	3.3	0.79	0.86
		CK	2.60	0.81	0.92	3.49	0.85	0.88	3.6	0.82	0.92

**Table 5:** Effects of water–fertilizer coupling on silage maize yield, water use efficiency and fertilizer partial productivity.

Year	Irrigation Method	Treatment	Yield (ton ha^−1^)	Water Use Efficiency (kg ha^−1^ mm^−1^)	Fertilizer Partial Productivity (kg·kg^−1^)
2023	Drip irrigation under mulch	MY1	47.3	192.76	106.58
		MY2	48.0	195.58	86.51
		MY3	48.7	198.40	73.14
		MK1	51.2	176.19	115.28
		MK2	66.2	227.71	119.19
		MK3	62.8	216.06	94.24
		MF1	59.5	177.34	134.01
		MF2	61.9	184.61	111.60
		MF3	61.1	182.07	91.72
		CK	63.8	190.03	56.67
	Shallow-buried drip irrigation	NY1	45.9	111.09	103.46
		NY2	47.6	115.16	85.80
		NY3	48.7	117.79	73.13
		NK1	50.7	110.55	114.16
		NK2	66.0	144.02	118.98
		NK3	62.1	135.38	93.20
		NF1	59.2	117.58	133.34
		NF2	61.2	121.45	110.18
		NF3	61.3	121.71	92.01
		CK	63.1	125.25	56.06
2024	Drip irrigation under mulch	MY1	47.8	194.70	107.66
		MY2	48.5	197.56	87.39
		MY3	49.2	200.41	73.87
		MK1	51.7	177.97	116.44
		MK2	67.5	232.36	121.62
		MK3	63.4	218.24	95.20
		MF1	60.1	179.14	135.36
		MF2	63.2	188.38	113.87
		NF3	61.7	183.90	92.64
		CK	64.4	191.95	57.24
	Shallow-buried drip irrigation	NY1	46.4	112.21	104.50
		NY2	48.1	116.32	86.67
		NY3	49.7	120.19	74.62
		NK1	51.2	111.67	115.32
		NK2	66.7	145.47	120.18
		NK3	62.7	136.75	94.14
		NF1	59.8	118.77	134.68
		NF2	62.4	123.93	112.43
		NF3	61.9	122.94	92.94
		CK	63.7	126.51	56.62

**Table 6:** Effects of water–fertilizer coupling on silage maize yield, water use efficiency and fertilizer partial productivity in different hydrological years.

Planting Methods	Different Hydrological Years	Treatment	Yield (ton ha^−1^)	Water Use Efficiency (kg ha^−1^ mm^−1^)	Fertilizer Partial Productivity (kg·kg^−1^)
Drip irrigation under film	Wet year (2012)	P1	66.7	128.00	150.23
		P2	70.4	135.10	126.85
		P3	68.9	132.22	103.45
	Normal year (1993)	P4	64.2	145.58	144.59
		P5	67.1	152.15	120.90
		P6	66.3	150.34	99.55
	Dry year (2000)	P7	46.8	127.94	105.41
		P8	48.9	133.68	88.11
		P9	47.6	130.13	71.47
Shallow-buried drip irrigation	Wet year (2012)	Z1	65.7	126.08	147.97
		Z2	68.5	131.45	123.42
		Z3	67.4	129.34	101.2
	Normal year (1993)	Z4	60.9	138.10	137.16
		Z5	65.8	149.21	118.56
		Z6	63.7	144.44	95.65
	Dry year (2000)	Z7	45.1	123.29	101.58
		Z8	46.8	127.94	84.32
		Z9	45.7	124.93	68.62

**Table 7:** Subsurface drip irrigation fertigation system design.

Treatment	Irrigation Quota/mm						Fertilization Amount/(kg·ha^−1^)		
	Seedling	Jointing	Large Bell Mouth	Heading and Flowering	Heading and Flowering	Pustulation	Bottom Fertilizer	Jointing	Large Bell Mouth
MY1	10	16	16	16	16	16	300	72	72
MY2	10	16	16	16	16	16	375	90	90
MY3	10	16	16	16	16	16	450	108	108
MK1	15	24	24	24	24	24	300	72	72
MK2	15	24	24	24	24	24	375	90	90
MK3	15	24	24	24	24	24	450	108	108
MF1	20	32	32	32	32	32	300	72	72
MF2	20	32	32	32	32	32	375	90	90
MF3	20	32	32	32	32	32	450	108	108
CK	60	60	60	0	0	0	750	375	0

**Table 8:** Shallow-buried drip irrigation water and fertilizer system design.

Treatment	Irrigation Quota/mm						Fertilization Amount/(kg·ha^−1^)		
	Seedling	Jointing	Large Bell Mouth	Heading and Flowering	Heading and Flowering	Pustulation	Bottom Fertilizer	Jointing	Large Bell Mouth
NY1	10	16	16	16	16	16	300	72	72
NY2	10	16	16	16	16	16	375	90	90
NY3	10	16	16	16	16	16	450	108	108
NK1	15	24	24	24	24	24	300	72	72
NK2	15	24	24	24	24	24	375	90	90
NK3	15	24	24	24	24	24	450	108	108
NF1	20	32	32	32	32	32	300	72	72
NF2	20	32	32	32	32	32	375	90	90
NF3	20	32	32	32	32	32	450	108	108
CK	60	60	60	0	0	0	750	375	0

M and N are with or without mulching; Y, K and F represent treatment with different fertilizers. 1, 2, and 3 are different water treatments.

**Table 9:** Soil physical properties.

Soil Depth/(cm)	Particle Distribution/%			Soil Bulk Density /(g cm^−3^)	Field Capacity /(%)	θs/(cm^3^ cm^−3^)	θr/(cm^3^ cm^−3^)	Soil Type
	Clay (<0.002 mm)	Powder (0.05~0.002 mm)	Sand (5~0.05 mm)					
0–10	12.58	32.14	55.28	1.44	36.8	41.0	0.15	Sandy Loam
10–20	14.25	26.58	59.17	1.46	36.2	40.5	0.16	Sandy Loam
20–40	13.37	36.29	50.34	1.40	35.1	37.6	0.16	Sandy Loam
40–60	6.52	2.63	90.85	1.56	29.2	36.5	0.09	Sand Soil
60–80	6.43	2.75	90.82	1.56	29.2	36.5	0.09	Sandy Soil

θs: percentage of saturated water content. θr: wilting coefficient.

**Table 11:** Subsurface drip irrigation fertigation system simulation scenario plan.

Hydrological Years	Treatment	Irrigation Quota/mm						Fertilization Amount/(kg·ha^−1^)		
		Seedling	Jointing	Large Bell Mouth	Heading and Flowering	Heading and Flowering	Pustulation	Bottom Fertilizer	Jointing	Large Bell Mouth
Wet year (2012)	P1	10	16	16	16	16	16	300	72	72
	P2	10	16	16	16	16	16	375	90	90
	P3	10	16	16	16	16	16	450	108	108
Normal year (1993)	P4	15	24	24	24	24	24	300	72	72
	P5	15	24	24	24	24	24	375	90	90
	P6	15	24	24	24	24	24	450	108	108
Dry year (2000)	P7	20	32	32	32	32	32	300	72	72
	P8	20	32	32	32	32	32	375	90	90
	P9	20	32	32	32	32	32	450	108	108

**Table 12:** Shallow drip irrigation water and fertilizer simulation scenario scheme.

Hydrological Years	Treatment	Irrigation Quota/mm						Fertilization Amount/(kg·ha^−1^)		
		Seedling	Jointing	Large Bell Mouth	Heading and Flowering	Heading and Flowering	Pustulation	Bottom Fertilizer	Jointing	Large Bell Mouth
Wet year (2012)	Z1	10	16	16	16	16	16	300	72	72
	Z2	10	16	16	16	16	16	375	90	90
	Z3	10	16	16	16	16	16	450	108	108
Normal year (1993)	Z4	15	24	24	24	24	24	300	72	72
	Z5	15	24	24	24	24	24	375	90	90
	Z6	15	24	24	24	24	24	450	108	108
Dry year (2000)	Z7	20	32	32	32	32	32	300	72	72
	Z8	20	32	32	32	32	32	375	90	90
	Z9	20	32	32	32	32	32	450	108	108

## Data Availability

The original contributions presented in this study are included in the article/Supplementary Material. Further inquiries can be directed to the corresponding author.

## Supplementary Materials

The following supporting information can be downloaded at: https://www.mdpi.com/article/10.3390/plants15020228/s1, Table S1. The measured value of aboveground biomass of drip irrigation under film (ton hm^−2^). Table S2. The measured value of aboveground biomass of shallow drip irrigation (ton hm^−2^). Table S3. The measured value of soil moisture content of drip irrigation under film (%). Table S4. The measured value of soil moisture content of shallow drip irrigation (%).
