# Supplementary material for: Research on Water and Fertilizer Use Strategies for Silage Corn Under Different Irrigation Methods to Mitigate Abiotic Stress
Source: Plants (Basel). 2026 Jan 11;15(2):228. doi: 10.3390/plants15020228 (PMC12844661; doi:10.3390/plants15020228)
Supplement: Supplementary file 1 [file plants-15-00228-s001.zip › plants-4054951-supplementary.pdf]

Table S1. The measured value of aboveground biomass of drip irrigation under film (ton hm^{-2})

Year	Aboveground biomass (ton hm^{-2})									
	MY1	MY2	MY3	MK1	MK2	MK3	MF1	MF2	MF3	CK
Drip irrigation under film (2024)	6.4	6.4	6.4	7.3	10.0	9.1	7.3	8.2	8.2	9.1
	93.6	96.4	100.0	104.5	141.8	130.0	112.7	125.5	120.0	133.6
	177.3	180.0	182.7	188.2	269.1	234.5	204.5	226.4	219.1	250.0
	234.5	239.1	244.5	250.0	374.5	333.6	276.4	310.9	305.5	350.0
	353.6	357.3	360.0	370.9	503.6	469.1	406.4	431.8	421.8	482.7
	419.1	426.4	431.8	442.7	583.6	541.8	491.8	516.4	508.2	567.3
	434.5	440.9	447.3	470.0	613.6	576.4	546.4	574.5	560.9	585.5
Drip irrigation under film (2023)	4.5	4.5	5.5	5.5	8.2	7.3	5.5	6.4	6.4	8.2
	73.6	76.4	78.2	80.9	98.2	83.6	82.7	89.1	86.4	95.5
	150.9	153.6	157.3	161.8	230.0	205.5	175.5	192.7	190.0	220.9
	205.5	210.0	216.4	223.6	337.3	313.6	264.5	294.5	288.2	329.1
	313.6	321.8	333.6	346.4	479.1	420.9	337.3	407.3	401.8	460.0
	411.8	419.1	425.5	435.5	560.9	511.8	486.4	500.9	492.7	542.7
	430.0	436.4	442.7	465.5	601.8	570.9	540.9	562.7	555.5	580.0

Table S2. The measured value of aboveground biomass of shallow drip irrigation (ton hm^{-2})

Year	Aboveground biomass (ton hm^{-2})									
	NY1	NY2	NY3	NK1	NK2	NK3	NF1	NF2	NF3	CK
Shallow buried drip irrigation (2024)	2.7	2.7	2.7	3.6	6.4	4.5	3.6	4.5	4.5	5.5
	23.6	24.5	25.5	29.1	38.2	34.5	31.8	34.5	32.7	37.3
	62.7	66.4	70.9	78.2	135.5	115.5	98.2	112.7	105.5	121.8
	163.6	169.1	171.8	183.6	269.1	242.7	208.2	237.3	225.5	253.6
	275.5	285.5	292.7	305.5	410.0	388.2	344.5	369.1	361.8	395.5
	353.6	364.5	388.2	398.2	540.9	496.4	450.0	483.6	467.3	517.3
	421.8	437.3	451.8	465.5	606.4	570.0	543.6	567.3	562.7	579.1
Shallow buried drip irrigation (2023)	3.6	3.6	3.6	4.5	7.3	5.5	4.5	5.5	5.5	6.4
	59.1	62.7	63.6	64.5	76.4	71.8	68.2	69.1	69.1	74.5
	116.4	120.9	131.8	140.0	189.1	171.8	150.0	158.2	164.5	178.2
	176.4	186.4	196.4	207.3	294.5	270.0	225.5	240.9	244.5	283.6
	264.5	270.0	296.4	316.4	431.8	389.1	337.3	360.9	369.1	415.5
	370.0	374.5	378.2	397.3	539.1	490.0	430.0	460.9	464.5	503.6
	417.3	432.7	442.7	460.9	600.0	564.5	538.2	556.4	557.3	573.6

Table S3. The measured value of soil moisture content of drip irrigation under film (%)

Year	Soil moisture content (%)									
	MY1	MY2	MY3	MK1	MK2	MK3	MF1	MF2	MF3	CK
Drip	17.2	21.3	23.5	18.6	19.8	22.5	17.9	20.5	21.8	22.5

irrigation under film (2024)	14.8	16.8	20.2	16.1	15.4	19.3	15.2	16.5	18.3	19.4
	12.3	13.4	16.9	11.5	14.1	15.3	11.9	13.4	15.8	16.6
	15.2	24.3	32.6	16.4	26.2	30.7	15.2	25.4	29.7	15.2
	11.4	19.8	23.7	12.6	20.8	24.5	11.6	19.1	22.5	12.8
	7.4	14.5	14.4	8.5	15.4	16.9	9.6	16.8	13.7	10.3
	15.7	20.2	21.7	15.2	21.7	19.5	16.7	19.6	21.3	26.5
	17.5	20.8	22.5	14.5	19.4	23.7	16.9	23.7	22.9	19.4
	14.3	15.7	14.6	15.7	12.2	13.4	11.1	16.4	14.6	11.5
	15.7	18.8	20.9	16.9	20.4	21.8	18.1	20.8	22.1	9.8
	10.3	12.4	13.8	12.9	11.8	14.1	13.6	14.3	15.5	29.5
	15.8	13.1	10.5	14.9	12.3	11.9	16.3	13.4	10.2	17.8
	23.4	17.8	18.9	24.1	16.9	19.5	23.2	17.6	18.5	20.1
	16.6	14.8	13.2	15.7	14.2	13.7	16.8	15.3	13.2	14.7
	14.2	15.7	18.5	13.6	14.2	17.9	14.5	15.3	18.6	27.4
	11.3	15.8	19.4	12.5	16.3	20.1	11.6	16.2	19.7	15.3
	16.5	21.5	26.1	15.2	22.6	25.8	16.4	21.7	26.1	10.2
Drip irrigation under film (2023)	17.9	18.6	22.8	18.3	21.7	23.4	18.2	19.7	21.7	23.1
	15.7	17.2	20.3	16.7	19.6	21	16.9	17.8	19.9	21.7
	13.8	15	17.2	14.4	17.3	18.4	15	15.8	17.2	18.7
	15.8	20.5	25.4	16.5	21.4	26.7	15.9	19.9	26.7	18.4
	12.4	18.6	23.5	15.2	17.8	24.9	13.8	17.7	24.9	17.6
	11.3	17.4	20.4	12.5	16.9	21.2	13.2	17.1	21.4	16.8
	16.7	21.2	22.3	14.7	17.1	21.7	14.8	16.9	21.9	25.1
	17.1	21.6	20.4	14.3	17.2	19.5	14.6	17.3	19.7	22.5
	15.4	18.7	16.2	13.1	15.4	16.6	13.5	15.8	15.3	19.8
	15.7	19.2	18.4	13.5	15.9	17.1	13.6	16.4	19.4	15.2
	14.6	16.8	17.5	12.6	13.7	15.3	12.9	15.3	18.1	24.8
	16.8	14.3	15.5	13.9	11.9	14.2	15.6	14.5	16.5	18.9
	22.9	15.4	17.2	23.7	16.8	18.6	23.5	16.8	18.3	20.4
	22.3	18.4	20.5	21.3	18.4	19.8	23.1	17.5	18.7	16.9
	17.3	14.7	19.4	18.5	14.1	19.9	16.9	13.8	18.6	22.3
	14.8	15.7	19.6	14.1	16.2	19.6	13.6	15.7	18.2	19.9
	15.3	17.9	21.5	15.9	18.5	22.4	13.7	18.4	24.8	16.7

Table S4. The measured value of soil moisture content of shallow drip irrigation (%)

Year	Soil moisture content（%）									
	NY1	NY2	NY3	NK1	NK2	NK3	NF1	NF2	NF3	CK
Shallow buried drip irrigation (2024)	16.4	19.3	21.5	17.8	20.1	22.0	17.7	19.0	20.5	21.3
	13.6	16.6	18.8	15.3	17.0	19.1	16.1	16.3	17.5	18.9
	12.5	14.2	15.8	12.7	15.4	16.4	13.8	14.0	15.2	16.8
	16.2	23.1	28.0	16.5	23.8	28.0	15.9	22.9	28.1	16.0
	12.3	18.2	22.0	13.8	18.5	22.9	12.5	18.1	22.4	14.3

	9.9	15.3	16.5	9.8	15.3	17.4	11.0	15.8	16.5	12.7
	17.4	22.0	23.3	16.3	20.7	22.8	16.7	20.2	23.5	25.2
	16.9	20.6	20.1	14.9	17.9	20.0	15.6	19.1	19.9	19.9
	13.8	15.7	13.5	13.1	13.5	13.5	11.8	14.9	13.2	15.3
	16.5	19.8	19.9	15.3	18.4	19.4	15.7	18.6	20.9	11.9
	14.0	15.3	15.4	14.0	13.9	14.5	14.6	15.3	16.3	27.7
	16.0	12.3	12.0	14.0	10.7	11.6	15.2	12.4	12.5	17.9
	23.4	17.9	18.3	24.2	18.2	19.6	23.5	19.1	18.5	20.7
	18.5	15.6	15.8	17.7	14.8	15.0	18.8	15.0	14.7	15.3
	16.4	19.3	21.5	17.8	20.1	22.0	17.7	19.0	20.5	21.3
	13.6	16.6	18.8	15.3	17.0	19.1	16.1	16.3	17.5	18.9
	12.5	14.2	15.8	12.7	15.4	16.4	13.8	14.0	15.2	16.8
Shallow buried drip irrigation (2023)	16.8	19.4	17.8	16.7	17.9	20.1	19.6	17.5	16.9	15.2
	12.1	15.2	14.9	11.2	12.5	15.4	15.7	12.6	11.7	14.5
	8.9	12.3	11.6	8.9	12.3	11.3	12.4	9.8	8.7	12.3
	18.2	27.3	29.5	15.1	26.4	28.6	17.6	25.9	28.6	12.1
	13.6	18.2	17.3	12.8	18.2	17.2	13.2	17.8	16.8	9.3
	10.4	13.2	9.4	6.3	12.8	11.4	10.3	12.4	10.6	7.7
	19.9	26.5	27.9	20.2	26.5	27.9	19.7	26.9	29.7	23.6
	16.2	17.8	17.5	15.9	18.2	16.5	16.6	18.3	16.8	16.2
	10.6	8.5	8.1	10.8	12.2	8.8	7.9	11.4	8.5	12.3
	17.1	21.3	22.1	16.5	21.9	20.7	16.2	18.2	21.9	8.6
	15.8	14.9	13.2	17.2	15.6	12.8	19.5	15.6	12.4	21.4
	13.7	8.3	6.7	12.9	8.1	6.9	13.1	8.9	7.1	15.4
	25.2	23.4	19.6	25.9	24.6	20.4	26.7	24.3	19.3	19.7
	12.3	7.3	6.7	12.9	8.3	7.2	13.2	10.1	7.8	11.9
	11.7	12.5	13.6	11.5	12.7	14.2	11.6	12.7	13.5	17.4
	12.8	16.4	20.1	10.2	15.2	20.4	13.2	16.3	20.1	11.5
	12.5	13.2	15.8	11.3	14.1	16.4	11.6	13.8	15.6	8.4
